# Biocompatible Calcium Ion-Doped Magnesium Ferrite Nanoparticles as a New Family of Photothermal Therapeutic Materials for Cancer Treatment

**DOI:** 10.3390/pharmaceutics15051555

**Published:** 2023-05-21

**Authors:** Panchanathan Manivasagan, Sekar Ashokkumar, Ala Manohar, Ara Joe, Hyo-Won Han, Sun-Hwa Seo, Thavasyappan Thambi, Hai-Sang Duong, Nagendra Kumar Kaushik, Ki Hyeon Kim, Eun Ha Choi, Eue-Soon Jang

**Affiliations:** 1 Department of Applied Chemistry, Kumoh National Institute of Technology, Daehak-ro 61, Gumi 39177, Republic of Korea; manimaribtech@gmail.com (P.M.); jar@kumoh.ac.kr (A.J.); 20101414hyowon@kumoh.ac.kr (H.-W.H.); 20126051@kumoh.ac.kr (S.-H.S.); 2 Plasma Bioscience Research Centre, Applied Plasma Medicine Center, Department of Electrical and Biological Physics, Kwangwoon University, Seoul 01897, Republic of Korea; kumarebt@kw.ac.kr (S.A.); kaushik.nagendra@kw.ac.kr (N.K.K.); 3 Department of Physics, Yeungnam University, Gyeongsan 38541, Republic of Korea; amanohar.svu@gmail.com (A.M.); kee1@ynu.ac.kr (K.H.K.); 4 School of Chemical Engineering, Theranostic Macromolecules Research Center, Sungkyunkwan University, Suwon 16419, Republic of Korea; thambi@skku.edu; 5 Faculty of Applied Sciences, Ton Duc Thang University, Ho Chi Minh City 700000, Vietnam; duonghaisang99@gmail.com

**Keywords:** ferrite nanoparticles, NIR laser, biocompatibility, photothermal therapy, cancer

## Abstract

Novel biocompatible and efficient photothermal (PT) therapeutic materials for cancer treatment have recently garnered significant attention, owing to their effective ablation of cancer cells, minimal invasiveness, quick recovery, and minimal damage to healthy cells. In this study, we designed and developed calcium ion-doped magnesium ferrite nanoparticles (Ca^2+^-doped MgFe_2_O_4_ NPs) as novel and effective PT therapeutic materials for cancer treatment, owing to their good biocompatibility, biosafety, high near-infrared (NIR) absorption, easy localization, short treatment period, remote controllability, high efficiency, and high specificity. The studied Ca^2+^-doped MgFe_2_O_4_ NPs exhibited a uniform spherical morphology with particle sizes of 14.24 ± 1.32 nm and a strong PT conversion efficiency (30.12%), making them promising for cancer photothermal therapy (PTT). In vitro experiments showed that Ca^2+^-doped MgFe_2_O_4_ NPs had no significant cytotoxic effects on non-laser-irradiated MDA-MB-231 cells, confirming that Ca^2+^-doped MgFe_2_O_4_ NPs exhibited high biocompatibility. More interestingly, Ca^2+^-doped MgFe_2_O_4_ NPs exhibited superior cytotoxicity to laser-irradiated MDA-MB-231 cells, inducing significant cell death. Our study proposes novel, safe, high-efficiency, and biocompatible PT therapeutics for treating cancers, opening new vistas for the future development of cancer PTT.

## 1. Introduction

Cancer remains one of the most challenging life- and health-threatening diseases worldwide [1,2]. Existing conventional cancer strategies, including chemotherapy, surgery, and radiotherapy, have strong side effects and suffer from cumulative radiation doses, high recurrence rates, complications, and low patient satisfaction [3]. Consequently, novel and patient-friendly therapeutic strategies are urgently needed to effectively treat cancers with high specificity [4]. Recently, owing to the rapid advancement of nanotechnology and nanoscience, many novel and emerging nanoparticle-based treatment strategies have been developed, such as photothermal therapy (PTT), radiation therapy, photocatalytic therapy, and ultrasound therapy [5,6,7,8,9]. PTT is a potential alternative to conventional cancer strategies, owing to its minimal invasiveness, short treatment period, easy localization, low cost, remote controllability, high efficiency, and high specificity [10]. During photothermal (PT) experiments, PT-conversion nanoparticles can absorb and convert near-infrared (NIR) light energy into heat energy to kill cancer cells [11]. Consequently, PTT is regarded as one of the most promising therapeutic strategies for treating cancers with minimal invasiveness [12,13,14]. 

To date, various nanoparticles, such as magnetic iron oxide nanomaterials [15,16], gold nanostructured materials [17,18], carbon nanomaterials [19], molybdenum sulfide [20], copper sulfide [21], and organic dye-based nanocarriers [22], have been developed as PT conversion materials for cancer treatment. Among these, magnetic iron oxide nanomaterials have recently attracted much interest in studies of drug delivery, hyperthermia, phototherapy, chemodynamic therapy, and magnetic resonance imaging (MRI) in various biomedical applications, owing to their excellent magnetic properties, low toxicity, and high stability [23,24]. Compared with magnetic iron oxide nanomaterials, superparamagnetic iron oxide nanoparticles such as calcium ion-doped magnesium ferrite nanoparticles (Ca^2+^-doped MgFe_2_O_4_ NPs), have an average particle size smaller than 20 nm, which is more suitable for clinical diagnosis and therapy [25,26]. Ca^2+^-doped MgFe_2_O_4_ NPs have been reported to exhibit excellent biocompatibility, stability, high heating efficiency, and good saturation magnetization, making them excellent candidates for hyperthermia, PTT, and chemodynamic therapy [27]. Ca^2+^-doped MgFe_2_O_4_ NPs have been widely demonstrated for more efficient and safe cancer treatment approaches, such as PTT [12]. In this study, we report novel biocompatible Ca^2+^-doped MgFe_2_O_4_ NPs as effective PT-ablation agents for cancer treatment.

## 2. Materials and Methods

### 2.1. Reagents 

All reagents and solvents were of the highest analytical grade, were obtained from Sigma-Aldrich, and were used as received without any further purification. 

### 2.2. Preparation of Ca^2+^-Doped MgFe_2_O_4_ NPs

Ca^2+^-doped MgFe_2_O_4_ NPs were synthesized based on the previously described solvothermal reflux (SR) method with slight modifications [28,29]. Briefly, benzyl ether (40 mL), oleic acid (9.0 mL), and oleylamine (11 mL) were mixed, stoichiometric metal precursors were added to the reaction solution, and the reaction solution was stirred for 10 min. The reaction solution was heated at 300 °C for 1 h and then, cooled to room temperature (RT). Finally, the reaction solution was separated by centrifugation and re-dispersed in *n*-hexane for further experiments. 

### 2.3. Characterization

The optical absorbance spectra of the prepared Ca^2+^-doped MgFe_2_O_4_ NPs were recorded using a UV–vis spectrophotometer (Shimadzu UV-2600). The particle size distribution of the prepared Ca^2+^-doped MgFe_2_O_4_ NPs was measured using dynamic light scattering (DLS) (Brookhaven Ins. Cor., 90 plus particle size analyzer). Fourier-transform infrared (FTIR) spectra of the prepared Ca^2+^-doped MgFe_2_O_4_ NPs were obtained as KBr pellets using a Thermo Scientific iD1 transmission spectrometer. Powder X-ray diffraction (XRD) analysis of the prepared Ca^2+^-doped MgFe_2_O_4_ NPs was performed using an X-ray diffractometer (Miniflex 600, Rigaku) with a Cu-Kα radiation source. The morphologies of the prepared Ca^2+^-doped MgFe_2_O_4_ NPs were investigated using a scanning electron microscope (SEM, JSM-7001F) with attached energy dispersive X-ray (EDX) mapping, transmission electron microscope (TEM, JEM 2100), and Cs-corrected field emission TEM (Cs-corrected FETEM, JEM-ARM200F) equipped with an EDX spectroscopy and mapping. X-ray photoelectron spectroscopy (XPS) was used to assess the compositional and chemical states (Kα, Thermo Fisher Scientific, Waltham, MA, USA). The stability of the prepared Ca^2+^-doped MgFe_2_O_4_ NPs was assessed for 30 days at RT in deionized water (DW), phosphate-buffered saline (PBS), and 10% fetal bovine serum (FBS), and was determined using a UV–vis spectrophotometer and TEM. For colloidal stability in Roswell Park Memorial Institute (RPMI) 1640 medium, the Ca^2+^-doped MgFe_2_O_4_ NPs were incubated in the RPMI medium supplemented with 10% FBS for various time intervals. The hydrodynamic size was measured using dynamic light scattering (DLS) (Brookhaven Ins. Cor., 90 plus particle size analyzer).

### 2.4. Determining the PT Effect of Ca^2+^-Doped MgFe_2_O_4_ NPs

Aqueous solutions of Ca^2+^-doped MgFe_2_O_4_ NPs at various concentrations (75, 100, and 125 µg/mL) were recorded using a UV–vis spectrophotometer. One milliliter of Ca^2+^-doped MgFe_2_O_4_ NPs aqueous solution in a quartz cuvette, with different concentrations (75, 100, and 125 µg/mL), was exposed to 785-nm-wavelength laser irradiation (CNI laser, China) at power densities of 0.5 W/cm^2^ and 0.8 W/cm^2^ for 300 s, and the solutions’ temperatures were monitored using a compact thermal camera (FLIR Cx-Series). The PT stability of the solution with the prepared Ca^2+^-doped MgFe_2_O_4_ NPs (125 µg/mL) in a quartz cuvette was assessed by exposing it to an NIR laser (785 nm, 0.8 W/cm^2^) for 300 s (NIR laser power turned on) and then allowing it to cool to RT for 900 s (NIR laser power turned off). The NIR laser power on/off cycles were applied four times to test the PT stability. The PT conversion efficiency (*η*) of the prepared Ca^2+^-doped MgFe_2_O_4_ NPs (125 µg/mL) aqueous solution was calculated according to the available reference Equation (1) [30,31,32]. The UV–vis spectra and TEM images of the Ca^2+^-doped MgFe_2_O_4_ NPs were observed after four laser on/off cycles.

(1) $$\eta =\frac{hS\left(T_{Max}-T_{Sur}\right)-Q_{dis}}{I\left(1-10^{-A_{785}}\;\right)}\;$$

### 2.5. Cell Culture

The normal human dermal fibroblast (HDF) cell line and human breast cancer cell line (MDA-MB-231 cells) were obtained from the Korean Cell Line Bank (Seoul, Republic of Korea). Cells were cultured and propagated in Dulbecco’s modified Eagle’s medium (DMEM) and Roswell Park Memorial Institute (RPMI) 1640 medium supplemented with 10% FBS and 1% penicillin/streptomycin at 37 °C in a humidified 5% CO_2_ atmosphere. 

### 2.6. In Vitro Cytotoxicity 

In vitro cytotoxicity was assessed using a standard 3-(4,5-dimethylthiazol-2-yl)-2,5-diphenyltetrazolium bromide (MTT) assay. The HDF and MDA-MB-231 cells (5 × 10^4^ cells/well) were cultured in 96-well flat-bottom plates in the DMEM and RPMI 1640 media. After 24-h-long incubation, Ca^2+^-doped MgFe_2_O_4_ NPs at various concentrations (range, 0–400 µg/mL) were added to 96-well plates for an additional 24 h and 48 h. Next, 100 µL of the MTT solution (0.5 mg/mL) was added to each well for an additional 4 h. The MTT-containing medium was removed, and dimethyl sulfoxide (DMSO, 100 µL) was added to all wells to dissolve the formazan crystals for 30 min. The absorbance of each well was recorded at 570 nm using a plate reader (Bio-TEK MQX200).

### 2.7. In Vitro Hemolysis Assay 

The in vitro hemolysis assay was performed according to the previously described method [33]. Red blood cells (RBCs) were collected from fresh mouse blood. Then, the RBCs were separated by centrifuging at 12,000 rpm for 7 min and diluted with 10 × PBS. Afterward, 0.3 µL of the RBC suspension was gently mixed with Ca^2+^-doped MgFe_2_O_4_ NPs in PBS at various concentrations (25, 50, 75, 100, and 125 µg/mL). RBC-treated PBS served as a negative control, whereas RBC-treated DW was used as a positive control. After incubation at RT for 4 h, all samples were centrifuged at 12,000 rpm for 7 min, and the absorbance of the supernatant was recorded at 541 nm using a microplate reader. 

### 2.8. Chick Chorioallantoic Membrane Assay

Freshly fertilized chicken eggs were obtained from chicken form (Chuncheon, Republic of Korea). Eggs were incubated at 37 °C and 60% relative humidity for 9 days. The chorioallantoic membrane (CAM) was removed on day 10 of the embryo development, to create a window in the eggshell. Then, 200 µL of PBS as a negative control, sodium hydroxide (NaOH, 0.1 M) as a positive control, and Ca^2+^-doped MgFe_2_O_4_ NPs (125 µg/mL) were added to all eggs and incubated for 5 min. Finally, toxicity was observed in terms of blood vessel damage and hemorrhage [34]. 

### 2.9. In Vitro PTT

For the in vitro PTT of cancer cells, MDA-MB-231 cells (5 × 10^4^ cells/well) were cultured in 96-well flat-bottom plates in the RPMI 1640 medium for 24 h. After that, Ca^2+^-doped MgFe_2_O_4_ NPs at various concentrations (range, 0–125 µg/mL) were added to 96-well plates for an additional 10 h incubation, following which the cells were irradiated with an NIR laser (785 nm, 0.8 W/cm^2^) for 300 s. After 5 h of additional incubation, cell viability was detected using the standard MTT assay, as previously described. In addition, cells were incubated with Ca^2+^-doped MgFe_2_O_4_ NPs (125 µg/mL) for 10 h, and then irradiated using an NIR laser (785 nm) at 0.5 W/cm^2^ and 0.8 W/cm^2^ for 300 s. After 5 h of additional incubation, cell viability was measured using the standard MTT method. 

For in vitro cell imaging, cells were cultured in 35-mm-diameter confocal dishes and incubated for 24 h at 37 °C. After that, cells were separated into four groups: control (100 µL PBS) only; control (100 µL PBS) + laser irradiation (0.8 W/cm^2^, 300 s); Ca^2+^-doped MgFe_2_O_4_ NPs (125 µg/mL) only; and Ca^2+^-doped MgFe_2_O_4_ NPs (125 µg/mL) + laser irradiation (0.8 W/cm^2^, 300 s). The cells were treated with PBS (100 µL) or Ca^2+^-doped MgFe_2_O_4_ NPs (125 µg/mL) for an additional 10 h incubation, following which some cells were irradiated using the NIR laser (785 nm, 0.8 W/cm^2^) for 300 s. After 5 h of incubation, cells were co-stained and tested using a live/dead staining assay with the fluorescent probes calcein-AM (green color) and propidium iodide (PI, red color), and also tested with trypan blue staining for 30 min. In addition, all cell nuclei and mitochondria were co-stained with Hoechst 33342 and MitoTracker Red for 30 min. Images of all groups of cells were acquired using a confocal laser scanning microscope (A1R; Nikon, Japan) in tail scan mode for live imaging. For the detection of reactive oxygen species (ROS), cells were cultured in a 35-mm-diameter culture plate and incubated for 24 h. After the incubation, all cells were divided into four groups, as previously described. All cells were stained with 2,7-dichlorofluorescein diacetate (DCFH-DA) for 30 min and imaged using a confocal fluorescence microscope in tail scan mode for live imaging. 

For the apoptosis assay, cells were seeded into 6-well flat-bottom plates in the RPMI 1640 medium for 24 h. The cells were then separated into four groups, as previously described. After various treatments, all cells were collected, washed with PBS, and co-stained with an Annexin V-FITC and PI staining kit (BD Pharmingen, Canada). Finally, the cells were analyzed using flow cytometry to detect apoptotic cells (BD FACSCalibur, Canada). 

Statistical analysis was performed, and all data were reported as the mean and standard error of the mean. Statistical differences were assessed using a one-way analysis of variance (ANOVA) test, using SPSS 14, with * denoting *p* < 0.05. 

## 3. Results and Discussion

### 3.1. Preparation and Characterization of Ca^2+^-Doped MgFe_2_O_4_ NPs

**Scheme 1:**
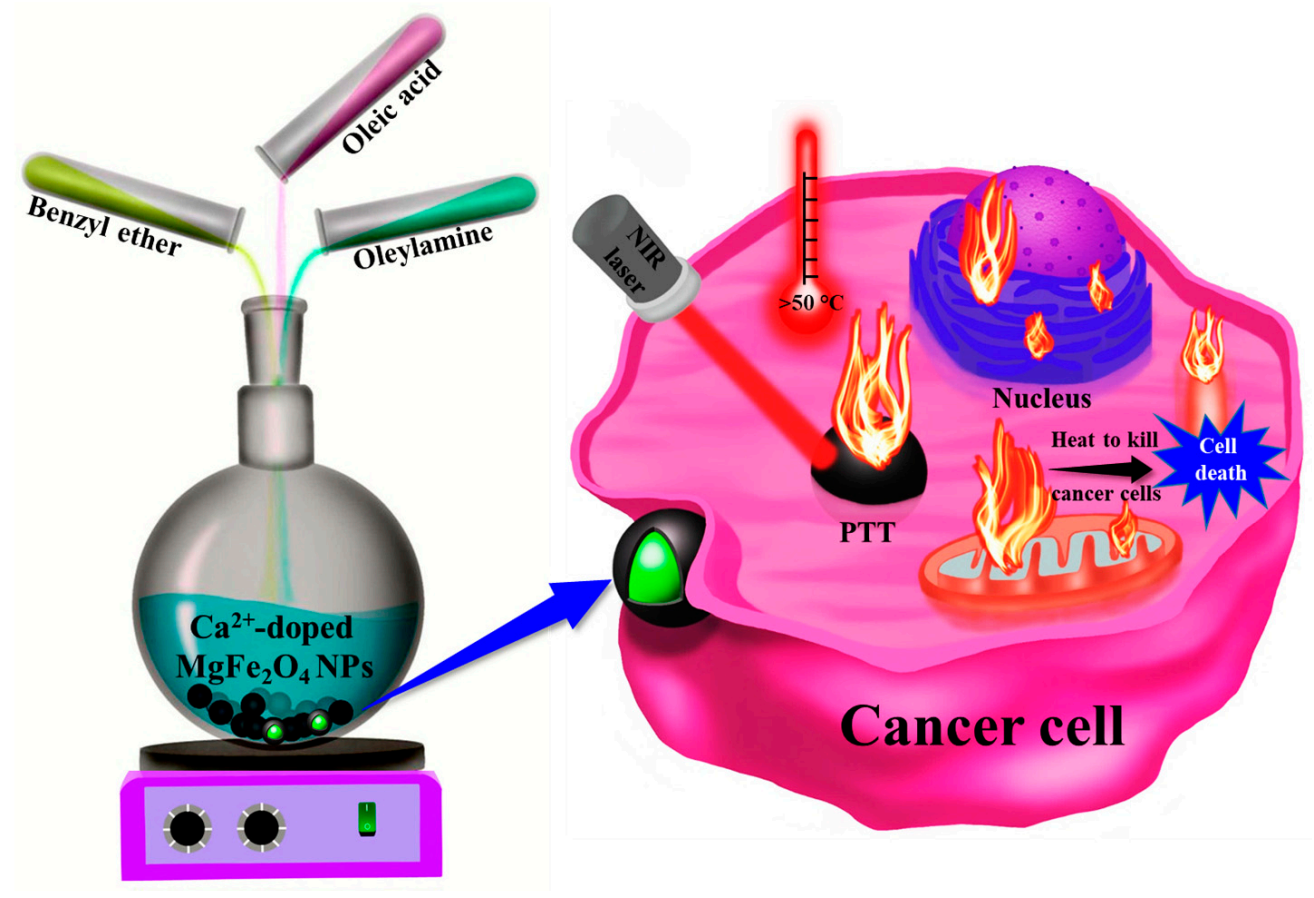
Ca^2+^-doped MgFe_2_O_4_ NPs were successfully prepared for PTT.

**Figure 1:**
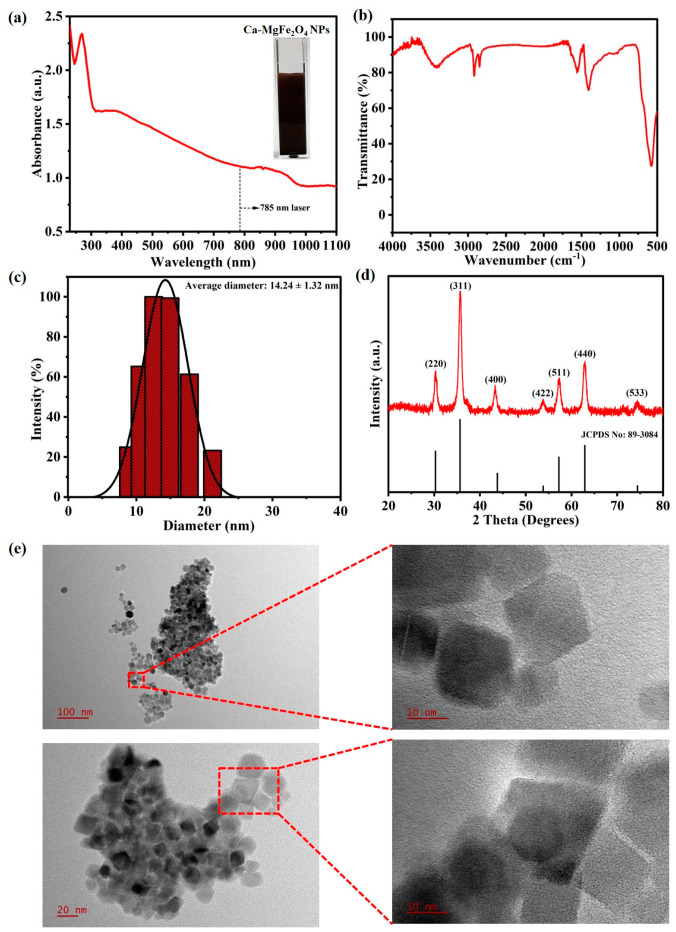
(**a**) UV–vis absorption spectra of Ca^2+^-doped MgFe_2_O_4_ NPs (the inset shows the Ca^2+^-doped MgFe_2_O_4_ NPs solution). (**b**) FTIR spectrum of Ca^2+^-doped MgFe_2_O_4_ NPs. (**c**) DLS measurements for determining the particle size distribution of Ca^2+^-doped MgFe_2_O_4_ NPs. (**d**) XRD analysis of Ca^2+^-doped MgFe_2_O_4_ NPs. (**e**) TEM images of Ca^2+^-doped MgFe_2_O_4_ NPs.

In this work, Ca^2+^-doped MgFe_2_O_4_ NPs were prepared using the solvothermal reflux technique at 300 °C for 1 h, as shown in Scheme 1 [28,29]. The UV–vis absorbance spectrum of the prepared Ca^2+^-doped MgFe_2_O_4_ NPs exhibited a broad absorbance in the ultraviolet to NIR range (200–1100 nm), indicating that Ca^2+^-doped MgFe_2_O_4_ NPs could be used as a potential PT material for PTT (Figure 1a). Figure 1b shows the FTIR spectra of Ca^2+^-doped MgFe_2_O_4_ NPs. Ca^2+^-doped MgFe_2_O_4_ NPs exhibited two major peaks at 579 and 1408 cm^−1^, which were attributed to the Fe–O and C=O bonds, while the absorption bands at 1558, 2847, and 2915 cm^−1^ were attributed to the C–H stretching vibration mode, confirming that the prepared Ca^2+^-doped MgFe_2_O_4_ NPs were successfully synthesized [35]. The size of the prepared Ca^2+^-doped MgFe_2_O_4_ NPs assessed using a DLS was 14.24 ± 1.32 nm, which is suitable for in vitro and in vivo therapeutic applications (Figure 1c). The Ca^2+^-doped MgFe_2_O_4_ NPs were analyzed by XRD (Figure 1d). The XRD pattern of the prepared Ca^2+^-doped MgFe_2_O_4_ NPs exhibited intense peaks corresponding to (220), (311), (400), (422), (511), (440), and (533), suggesting their crystalline nature [36]. All of the diffraction peaks matched well with the international standard data card Joint Committee on Powder Diffraction Standards (JCPDS) number 89-3084. 

**Figure 2:**
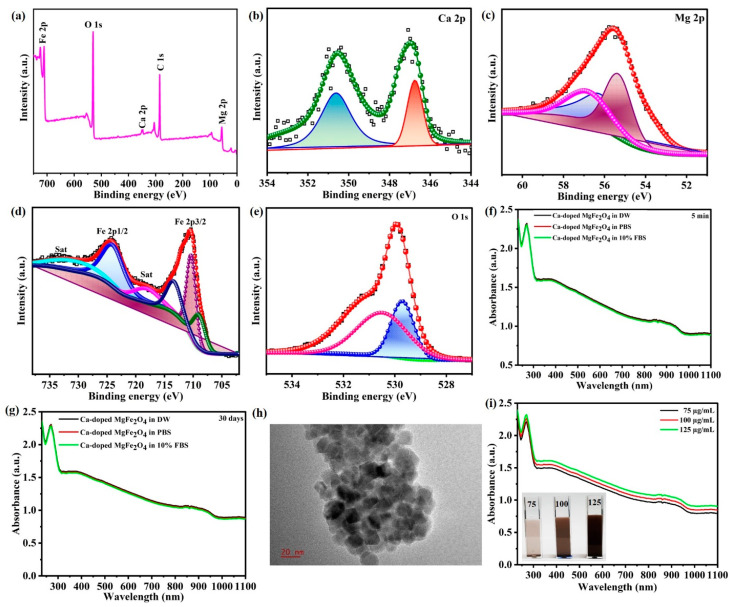
(**a**) XPS survey spectrum of Ca^2+^-doped MgFe_2_O_4_ NPs. XPS spectra for Ca 2p (**b**), Mg 2p (**c**), Fe 2p (**d**), and O 1s (**e**) of Ca^2+^-doped MgFe_2_O_4_ NPs. UV–vis absorption spectra of Ca^2+^-doped MgFe_2_O_4_ NPs in DW, PBS, and 10% FBS for 5 min (**f**) and 30 days (**g**). (**h**) TEM images of Ca^2+^-doped MgFe_2_O_4_ NPs in 10% FBS after 30 days. (**i**) UV–vis absorption spectra of Ca^2+^-doped MgFe_2_O_4_ NPs at various concentrations.

The morphology of the Ca^2+^-doped MgFe_2_O_4_ NPs was investigated using TEM and Cs-corrected FETEM with EDX mapping and EDX spectroscopy. The TEM images of Ca^2+^-doped MgFe_2_O_4_ NPs revealed that most of the particles were a nearly uniform and cube-like shape, with an average particle size of 14.24 ± 1.32 nm (Figure 1e and Figure S1). Our TEM images are similar to those in a previous report by Heiba et al. [37] and Sivagurunathan et al. [38], which showed cube-shaped ferrite NPs. The Cs-corrected FETEM with EDX mapping (Figure S2) and EDX spectroscopy (Figure S3) of Ca^2+^-doped MgFe_2_O_4_ NPs revealed the presence of Mg, Fe, Ca, and O. In addition, the SEM images of Ca^2+^-doped MgFe_2_O_4_ NPs showed that the particles were almost cube shape and had uniform morphologies (Figure S4). Similar SEM images are observed by Naaz et al. [39] for the cube shape of MgFe2O4 NPs. SEM-related EDX mapping (Figure S5) and EDX spectroscopy (Figure S6) revealed the presence of Mg, Fe, Ca, and O. XPS is a versatile surface analysis technique that is frequently used to examine compositional and chemical states [40]. The goal of the XPS analysis was to learn more about the valence states of the elements on the Ca^2+^-doped MgFe_2_O_4_ NPs surface. Survey scan spectra of the prepared nanoparticles (Ca, Mg, Fe, and O elements) are shown in Figure 2a. According to Figure 2b, the Ca 2p core is divided into 2p3/2 (binding energy = 346.91 eV) and 2p1/2 (binding energy = 350.52 eV) peaks [41]. The Mg^2+^ ions were assigned one characteristic peak at 55.59 eV and three deconvoluted peaks at 56.99, 56.36, and 55.41 eV, as shown in the Mg 2p spectra (Figure 2c). The XPS spectra of the Fe 2P core in the Fe^3+^ valence state are shown in Figure 2d. The estimated binding energies of the Fe 2p3/2 and Fe 2p1/2 lines were 710.53 eV and 724.17 eV, respectively. Fe^3+^ was predominant, as evidenced by the satellite peaks of the Fe 2p core at 732.45 and 718.33 eV [28]. The O1s signal is shown in Figure 2e, revealing that the main peaks were located at 530.64 and 529.72 eV. The former peak was equivalent to the surface-located lattice oxygen [28]. The long-term stability of Ca^2+^-doped MgFe_2_O_4_ NPs dispersions is essential for biomedical applications [42]. The stability test of the Ca^2+^-doped MgFe_2_O_4_ NPs dispersed in DW, PBS, and 10% FBS for 30 days was performed using a UV–vis spectrophotometer. As shown in Figure 2f,g, no obvious changes were observed in the UV–vis absorbance spectra of the Ca^2+^-doped MgFe_2_O_4_ NPs solution in DW, PBS, and 10% FBS for 30 days, implying that the prepared Ca^2+^-doped MgFe_2_O_4_ NPs exhibited good stability and high biocompatibility. The TEM image of Ca^2+^-doped MgFe_2_O_4_ NPs revealed a spherical morphology with uniform shapes in 10% FBS after 30 days (Figure 2h). For colloidal stability in the RPMI medium, the Ca^2+^-doped MgFe_2_O_4_ NPs were incubated in the RPMI medium with 10% FBS to assess their colloidal stability in biological systems. The hydrodynamic size of the Ca^2+^-doped MgFe_2_O_4_ NPs in RPMI medium was gradually increased because of small aggregates or precipitates in biological systems in the presence of amino acids and salts (Figure S7). Similar behavior has been reported for colloidal stability in the biological medium in the presence of NPs [43]. 

### 3.2. Determining the PT Effect of Ca^2+^-Doped MgFe_2_O_4_ NPs

**Figure 3:**
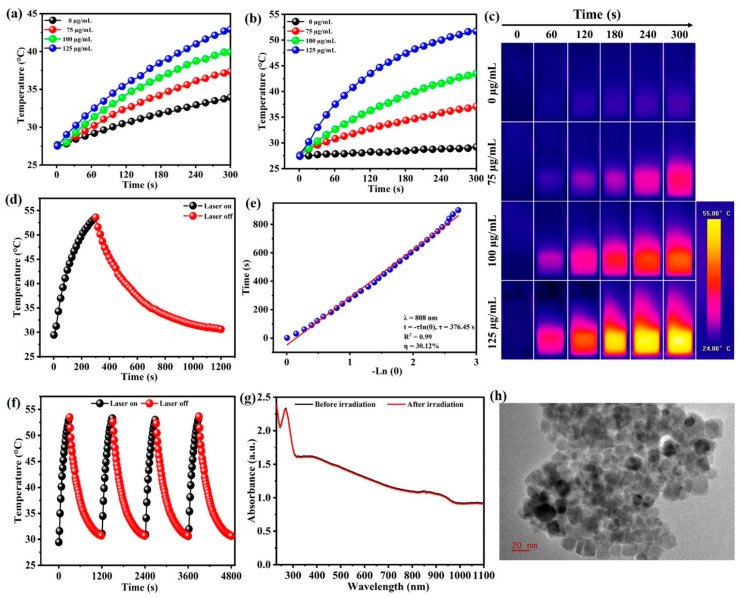
Temperature profiles for various concentrations of Ca^2+^-doped MgFe_2_O_4_ NPs irradiated with a 785 nm laser at 0.5 (**a**) and 0.8 W/cm^2^ (**b**) for 300 s. (**c**) Infrared thermal images for various concentrations of Ca^2+^-doped MgFe_2_O_4_ NPs irradiated with a 785 nm laser at 0.8 W/cm^2^ for 300 s. (**d**) Temperature profile of Ca^2+^-doped MgFe_2_O_4_ NPs (125 µg/mL) irradiated with a 785 nm laser at 0.8 W/cm^2^ for 300 s and then allowed to cool down to RT for 900 s. (**e**) A linear relationship between time and -Ln *θ* was found during the cooling period after the laser irradiation was turned off. (**f**) PT stability of Ca^2+^-doped MgFe_2_O_4_ NPs. (**g**) UV–vis absorption spectra of Ca^2+^-doped MgFe_2_O_4_ NPs in DW, before and after four on/off laser cycles. (**h**) TEM image of Ca^2+^-doped MgFe_2_O_4_ NPs after four on/off laser cycles.

The aqueous solutions of Ca^2+^-doped MgFe_2_O_4_ NPs at different concentrations (75, 100, and 125 µg/mL) were observed using a UV–vis spectrophotometer, revealing good absorption in the NIR range, suggesting that Ca^2+^-doped MgFe_2_O_4_ NPs are highly effective for PT-based cancer treatments (Figure 2i). To explore the PT effect of Ca^2+^-doped MgFe_2_O_4_ NPs, 1-mL-aqueous solutions of Ca^2+^-doped MgFe_2_O_4_ NPs in a quartz cuvette at various concentrations (75, 100, and 125 µg/mL) were exposed to NIR laser irradiation (785 nm) at power densities of 0.5 (Figure 3a) and 0.8 W/cm^2^ (Figure 3b) for 300 s, and their temperatures were recorded using a compact thermal imaging camera. The temperature of the Ca^2+^-doped MgFe_2_O_4_ NPs solution (125 µg/mL) increased to 51.7 °C within 300 s of the NIR laser irradiation at 0.8 W/cm^2^, and real-time temperature changes of Ca^2+^-doped MgFe_2_O_4_ NPs were captured using a compact thermal imaging camera (Figure 3c), whereas water (0 µg/mL) in the same treatment conditions only exhibited a slight temperature increase (from 27.56 °C to 29.2 °C). Therefore, Ca^2+^-doped MgFe_2_O_4_ NPs are excellent candidates for NIR-mediated PTT. The temperature change depended on the concentration, laser power density, and irradiation time [44]. 

To investigate the PT conversion efficiency of Ca^2+^-doped MgFe_2_O_4_ NPs, the temperature changes in the aqueous solution of Ca^2+^-doped MgFe_2_O_4_ NPs (125 µg/mL) were monitored in response to the 5 min 785-nm-wavelength laser irradiation at 0.8 W/cm^2^. After turning off the laser, the aqueous solution of Ca^2+^-doped MgFe_2_O_4_ NPs was allowed to slowly cool down to RT for 15 min during each cycle (Figure 3d). The estimated PT conversion efficiency (*η*) of the solution of Ca^2+^-doped MgFe_2_O_4_ NPs (125 µg/mL) was 30.12%, and the estimated time constant for the heat transfer of Ca^2+^-doped MgFe_2_O_4_ NPs was 376.45 s (Figure 3e), indicating that Ca^2+^-doped MgFe_2_O_4_ NPs can be used as an efficient PT material. In fact, the PT conversion efficiency of Ca^2+^-doped MgFe_2_O_4_ NPs was higher than previously reported classic photothermal agents such as gold nanorods (21%) [45], MoS_2_ nanosheets (24.4%) [46], Cu2-xS nanocrystals (16.3%) [47], and SiO2/Au nanoshells (30%) [48]. To further examine the PT stability of Ca^2+^-doped MgFe_2_O_4_ NPs, the aqueous solution of Ca^2+^-doped MgFe_2_O_4_ NPs (125 µg/mL) was exposed to the NIR laser irradiation (785 nm, 0.8 W/cm^2^) for 300 s, following which the laser was switched off for 900 s. After four on/off NIR laser cycles, the temperature profile of Ca^2+^-doped MgFe_2_O_4_ NPs exhibited no significant changes, indicating that the prepared Ca^2+^-doped MgFe_2_O_4_ NPs had high PT stability (Figure 3f). Furthermore, the optical properties of the aqueous solution of Ca^2+^-doped MgFe_2_O_4_ NPs were observed by UV–vis spectrophotometry after four on/off laser cycles, and revealed no noticeable changes in the absorbance spectrum (Figure 3g). The TEM image was also clearly visible after four on/off NIR laser cycles, revealing spherical particles with uniform shapes (Figure 3h). 

### 3.3. In Vitro Cytotoxicity, Hemolysis, and CAM Assay

**Figure 4:**
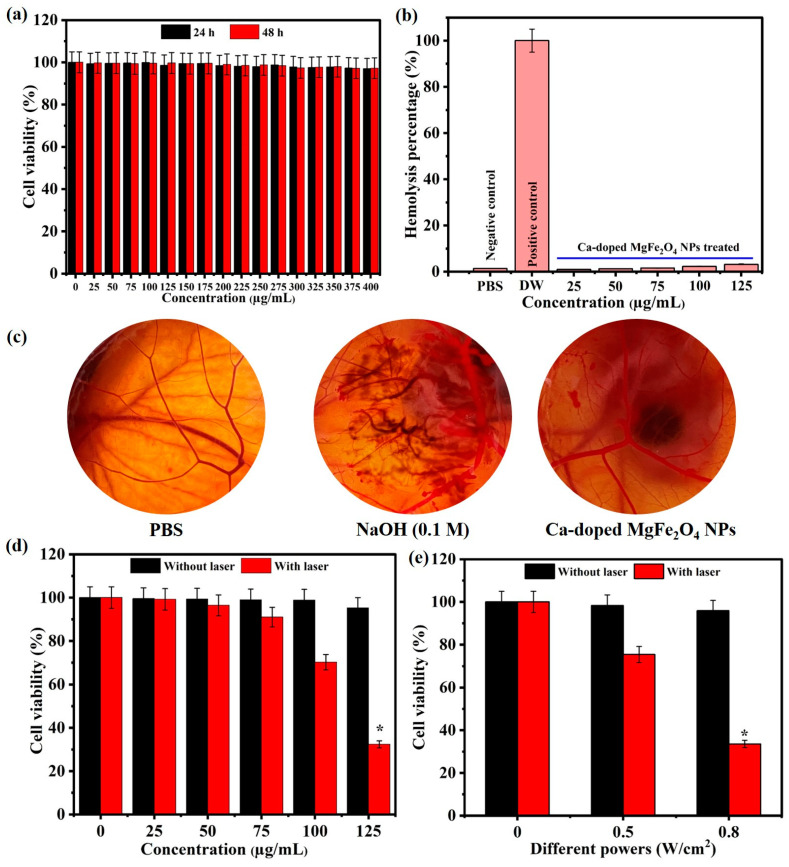
(**a**) In vitro biocompatibility of Ca^2+^-doped MgFe_2_O_4_ NPs at various concentrations on MDA-MB-231 cells after 24 h and 48 h. (**b**) In vitro hemolytic activity of Ca^2+^-doped MgFe_2_O_4_ NPs at various concentrations. (**c**) Chick CAM assay. (**d**) In vitro cell viability of Ca^2+^-doped MgFe_2_O_4_ NPs at various concentrations, assessed on treated cells that were laser-irradiated at 0.8 W/cm^2^ for 300 s. (**e**) In vitro cell viability of Ca^2+^-doped MgFe_2_O_4_ NPs (125 µg/mL), assessed on treated cells that were laser-irradiated at 0.5 and 0.8 W/cm^2^ for 300 s. The shown data are expressed as mean ± s.d., (*n* = 3), * *p* < 0.05.

The basic requirements for biomedical applications of NPs are high biocompatibility and safety, which are essential for industrializing nanomedical systems [49,50]. The in vitro cytotoxicity of Ca^2+^-doped MgFe_2_O_4_ NPs at various concentrations (0–400 µg/mL) for HDF cells and MDA-MB-231 cells was assessed using the MTT assay. As shown in Figure S8 and Figure 4a, Ca^2+^-doped MgFe_2_O_4_ NPs exhibited no noticeable cytotoxicity after 24 h and 48 h of incubation, for concentrations in the 0–400 µg/mL range, and the cell viability remained above 95%, implying that Ca^2+^-doped MgFe_2_O_4_ NPs had excellent biocompatibility. To investigate the hemocompatibility of Ca^2+^-doped MgFe_2_O_4_ NPs, a hemolysis assay was performed to explore the effect of Ca^2+^-doped MgFe_2_O_4_ NPs in PBS at various concentrations (25, 50, 75, 100, and 125 µg/mL) on RBCs. As shown in Figure 4b, the prepared Ca^2+^-doped MgFe_2_O_4_ NPs (125 µg/mL) demonstrated an incredibly low hemolysis ratio (3.2%), which was lower than the permissible limit (5%), confirming that Ca^2+^-doped MgFe_2_O_4_ NPs had good hemocompatibility and could be used for in vivo cancer treatments [51]. In addition, the toxicity of Ca^2+^-doped MgFe_2_O_4_ NPs (125 µg/mL) was evaluated using the chick CAM assay (Figure 4c). Ca^2+^-doped MgFe_2_O_4_ NP-treated chick embryos exhibited no observable toxicity in blood coagulation compared with PBS, indicating that Ca^2+^-doped MgFe_2_O_4_ NPs were non-toxic to egg CAM [34,52]. 

### 3.4. In Vitro PTT 

To investigate the PT effect of the prepared Ca^2+^-doped MgFe_2_O_4_ NPs, the in vitro PT effect was evaluated using the MTT assay. Cells were incubated with Ca^2+^-doped MgFe_2_O_4_ NPs at different concentrations (0–125 µg/mL) for 10 h and then irradiated using the NIR laser (785 nm, 0.8 W/cm^2^) for 300 s. As shown in Figure 4d, the viability of non-laser-irradiated cells was 94.5% at the high concentration of Ca^2+^-doped MgFe_2_O_4_ NPs (125 µg/mL), implying no observable effect on cancer cells. Most of the cells were killed after incubation with Ca^2+^-doped MgFe_2_O_4_ NPs (125 µg/mL) and irradiation using the NIR laser (785 nm, 0.8 W/cm^2^) for 300 s, and the cell viability decreased to 32.37%, confirming that laser-irradiated Ca^2+^-doped MgFe_2_O_4_ NPs induced significantly more cell death. Similar results were obtained when Ca^2+^-doped MgFe_2_O_4_ NPs were compared with various ferrite NPs (Table S1). In addition, cells were treated with Ca^2+^-doped MgFe_2_O_4_ NPs (125 µg/mL) for 10 h and irradiated using the NIR laser (785 nm) at 0.5 and 0.8 W/cm^2^ for 300 s (Figure 4e). Most of the cells were killed owing to the Ca^2+^-doped MgFe_2_O_4_ NPs (125 µg/mL) after being NIR laser-irradiated (0.8 W/cm^2^) for 300 s, demonstrating the promise of Ca^2+^-doped MgFe_2_O_4_ NPs as effective PT materials for treating cancers. 

**Figure 5:**
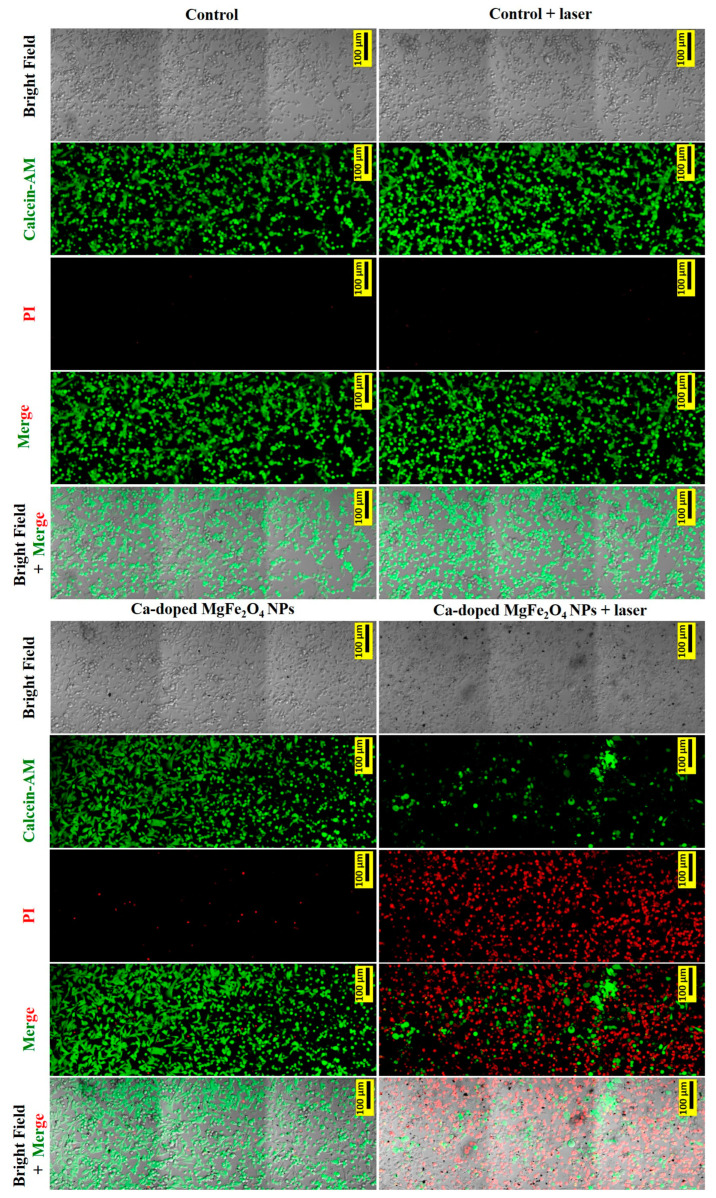
Confocal laser scanning microscope images of tile scans of MDA-MB-231 cells treated with PBS only, PBS + laser irradiation, Ca^2+^-doped MgFe_2_O_4_ NPs only, and Ca^2+^-doped MgFe_2_O_4_ NPs + laser irradiation. Calcein-AM (green) and PI (red) were used to distinguish between live and dead cells (20× magnification; scale bar: 100 µm).

Inspired by the above experiment, we further confirmed the results of the live/dead cell-staining calcein-AM/PI assay (Figure 5 and Figure S9). The MDA-MB-231 cells treated with Ca^2+^-doped MgFe_2_O_4_ NPs (125 µg/mL) were clearly evident in the presence of strong red fluorescence after the NIR laser irradiation, implying that more dead cells were detected in this group. In contrast, all the other groups exhibited intense green fluorescence and negligible cell death, indicating that more live cells were detected in the other groups. In addition, the live/dead staining assay was further confirmed by trypan blue staining, owing to their ability to distinguish between live (unstained) and dead (blue-stained) cells. As shown in Figure S10, no obvious changes were observed in the control cells with or without the NIR laser radiation, indicating that all the cells exhibited good morphology. The MDA-MB-231 cells that were treated with Ca^2+^-doped MgFe_2_O_4_ NPs (125 µg/mL) and were not laser-irradiated exhibited negligible cell death. In contrast, cells incubated with Ca^2+^-doped MgFe_2_O_4_ NPs (125 µg/mL) exhibited clear blue staining after the laser irradiation, indicating that more dead cells were present in this group. 

**Figure 6:**
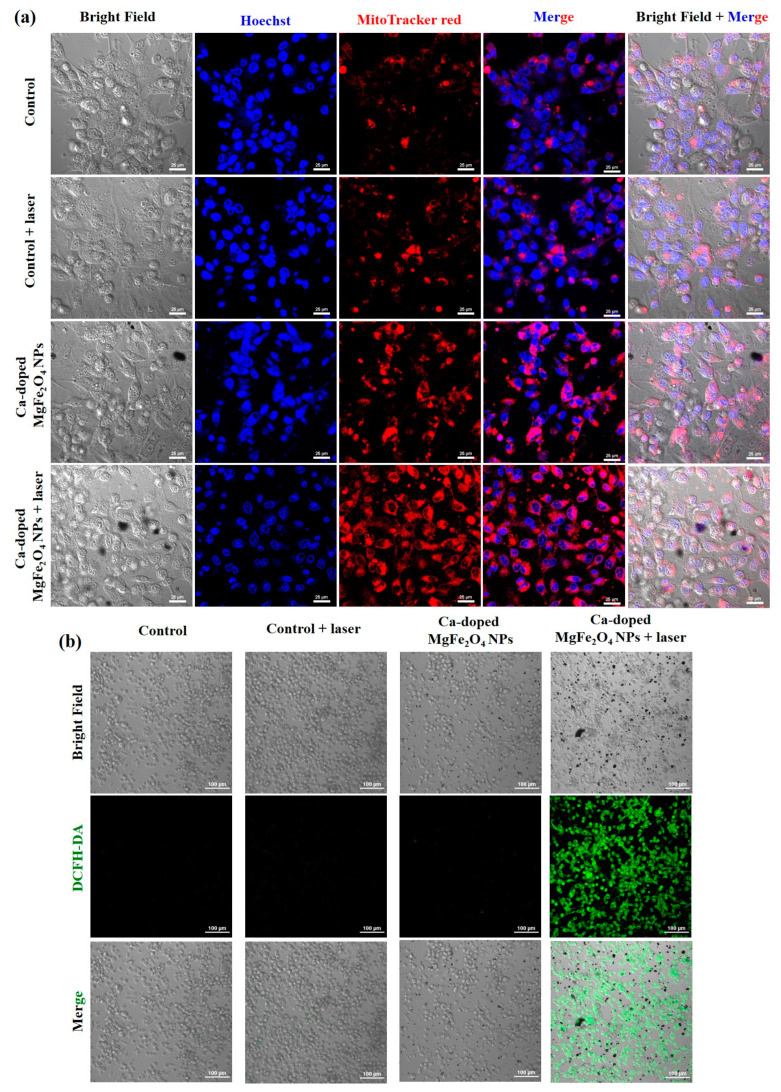
(**a**) Confocal laser scanning microscope images of MDA-MB-231 cells treated with PBS only, PBS + laser irradiation, Ca^2+^-doped MgFe_2_O_4_ NPs only, and Ca^2+^-doped MgFe_2_O_4_ NPs + laser irradiation. Hoechst 33342 and MitoTracker red were used to stain the nuclei and mitochondria (20× magnification; scale bar: 100 µm). (**b**) Confocal laser scanning microscope images of DCFH-DA-stained MDA-MB-231 cells treated with PBS only, PBS + laser irradiation, Ca^2+^-doped MgFe_2_O_4_ NPs only, and Ca^2+^-doped MgFe_2_O_4_ NPs + laser irradiation, for determining the intracellular ROS level (20× magnification; scale bar: 100 µm).

**Figure 7:**
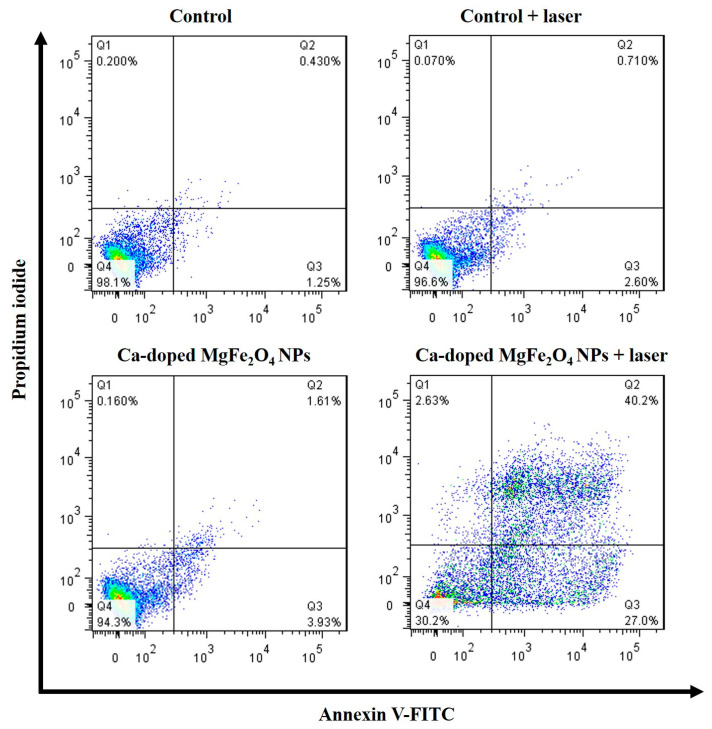
Flow cytometry analysis of the apoptosis of MDA-MB-231 cells, induced by PBS only, PBS + laser irradiation, Ca^2+^-doped MgFe_2_O_4_ NPs only, and Ca^2+^-doped MgFe_2_O_4_ NPs + laser irradiation using Annexin-V/PI staining.

In addition, each group of cells was further co-stained with Hoechst 33342 and MitoTracker Red, and evaluated using a confocal fluorescence microscope (Figure 6a). Hoechst 33342 and MitoTracker Red were used for nuclear and mitochondrial staining with blue and red fluorescence, respectively [53]. Hoechst 33342 clearly detected fragmented nuclei in the laser-irradiated Ca^2+^-doped MgFe_2_O_4_ NPs (125 µg/mL), and MitroTracker Red was colocalized with the mitochondria-specific marker, whereas the other groups showed that the nucleus and mitochondria appeared under normal conditions. The presence of toxic ROS in the MDA-MB-231 cells was assessed using DCFH-DA as a fluorescent dye, which could be converted into a potent green fluorescent molecule DCF in the presence of toxic ROS [35]. As shown in Figure 6b and Figure S11, the strong green fluorescence signal was almost undetectable in the control only, control + laser-irradiated, and Ca^2+^-doped MgFe_2_O_4_ NPs (125 µg/mL), which did not affect cancer cells. In contrast, Ca^2+^-doped MgFe_2_O_4_ NPs (125 µg/mL) caused strong fluorescence signals after the laser irradiation, which could lead to the production of ROS and damage biomolecules, mitochondria, and nuclei [54]. To evaluate the cell death mode after PT, cell apoptosis was also evaluated using flow cytometry (Figure 7). Ca^2+^-doped MgFe_2_O_4_ NPs (125 µg/mL) induced significant apoptosis (67.2%) after the laser irradiation, compared with the other groups (1.68%, 3.31%, and 5.54%), suggesting that the PT effect of Ca^2+^-doped MgFe_2_O_4_ NPs on MDA-MB-231 cells was enhanced by the NIR laser irradiation [55]. 

## 4. Conclusions

In this study, we successfully designed and developed novel biocompatible PT materials for cancer therapy. Ca^2+^-doped MgFe_2_O_4_ NPs were prepared using the solvothermal reflux method. The prepared Ca^2+^-doped MgFe_2_O_4_ NPs had a uniform and spherical morphology, with an average particle size of 14.24 ± 1.32 nm, and exhibited a strong PT conversion efficiency (30.12%), high biocompatibility, PT stability, high NIR absorption, and biosafety. Impressively, in vitro assays indicated that Ca^2+^-doped MgFe_2_O_4_ NPs did not exhibit any significant cytotoxicity against MDA-MB-231 cells without laser irradiation, implying that Ca^2+^-doped MgFe_2_O_4_ NPs had excellent biocompatibility. Cells treated with Ca^2+^-doped MgFe_2_O_4_ NPs exhibited a considerable PT ablation effect on NIR-laser-irradiated MDA-MB-231 cells, because these nanomaterials had good PT conversion efficiency, implying that laser-irradiated Ca^2+^-doped MgFe_2_O_4_ NPs induced significantly more cell death. To the best of our knowledge, there have been no prior studies on Ca^2+^-doped MgFe_2_O_4_ NPs as novel PT ablation materials for cancer treatment. The Ca^2+^-doped MgFe_2_O_4_ NPs developed in this study are promising as safe and efficient PT materials for cancer therapy in the near future. 

## Data Availability

Not applicable.

## Supplementary Materials

The following supporting information can be downloaded at: https://www.mdpi.com/article/10.3390/pharmaceutics15051555/s1, Figure S1: TEM image of Ca^2+^-doped MgFe_2_O_4_ NPs. Figure S2: Cs-corrected FETEM with EDX mapping. Figure S3: Cs-corrected FETEM with EDX spectroscopy. Figure S4: SEM images of Ca^2+^-doped MgFe_2_O_4_ NPs. Figure S5. SEM with EDX mapping of Ca^2+^-doped MgFe_2_O_4_ NPs. Figure S6: SEM-EDX spectra of Ca^2+^-doped MgFe_2_O_4_ NPs. Figure S7: Hydrodynamic size of Ca^2+^-doped MgFe_2_O_4_ NPs after incubation in RPMI medium supplemented with 10% FBS. Figure S8: In vitro biocompatibility of Ca^2+^-doped MgFe_2_O_4_ NPs at various concentrations on HDF cells after 24 h and 48 h. Figure S9: Confocal laser scanning microscope images of tile scans of MDA-MB-231 cells treated with PBS only, PBS + laser irradiation, Ca^2+^-doped MgFe_2_O_4_ NPs only, and Ca^2+^-doped MgFe_2_O_4_ NPs + laser irradiation. Calcein-AM (green) and PI (red) were used to distinguish between live and dead cells (20× magnification; scale bar: 100 µm). Figure S10. Optical images of trypan blue-stained MDA-MB-231 cells treated with PBS only, PBS + laser irradiation, Ca^2+^-doped MgFe_2_O_4_ NPs only, and Ca^2+^-doped MgFe_2_O_4_ NPs + laser irradiation (20× magnification; scale bar: 100 µm). Figure S11. Confocal laser scanning microscopy images of tile scans of DCFH-DA-stained MDA-MB-231 cells treated with PBS only, PBS + laser irradiation, Ca^2+^-doped MgFe_2_O_4_ NPs only, and Ca^2+^-doped MgFe_2_O_4_ NPs + laser irradiation, for determining the intracellular ROS level (20× magnification; scale bar: 100 µm). Table S1. Comparative study of the photothermal effect of Ca^2+^-doped MgFe_2_O_4_ NPs and various ferrite NPs. References [35,56,57,58] are cited in the supplementary materials.
