# Supplementary material for: Biocompatible Calcium Ion-Doped Magnesium Ferrite Nanoparticles as a New Family of Photothermal Therapeutic Materials for Cancer Treatment
Source: Pharmaceutics. 2023 May 21;15(5):1555. doi: 10.3390/pharmaceutics15051555 (PMC10221417; doi:10.3390/pharmaceutics15051555)
Supplement: Supplementary file 1 [file pharmaceutics-15-01555-s001.zip › pharmaceutics-2361510-supplementary.pdf]

Biocompatible calcium ion-doped magnesium ferrite nanoparticles as a new family of photothermal therapeutic materials for cancer treatment

Panchanathan Manivasagan [1†], Sekar Ashokkumar [2†], Ala Manohar [3†], Ara Joe [1], Hyo-Won Han [1], Sun-Hwa Seo [1], Thavasyappan Thambi [4], Hai-Sang Duong [5], Nagendra Kumar Kaushik [2], Ki Hyeon Kim [3], Eun Ha Choi [2,*], and Eue-Soon Jang [1,*]

[1] Department of Applied Chemistry, Kumoh National Institute of Technology, Daehak-ro 61, Gumi, Gyeongbuk 39177, Republic of Korea; manimaribtech@gmail.com (P. M.); jar@kumoh.ac.kr (A.J.); 20101414hyowon@kumoh.ac.kr (H.-W.H.); 20126051@kumoh.ac.kr (S.-H. S.)

[2] Plasma Bioscience Research Centre, Applied Plasma Medicine Center, Department of Electrical and Biological Physics, Kwangwoon University, Seoul 01897, Republic of Korea; kumarebt@kw.ac.kr (S.A.); kaushik.nagendra@kw.ac.kr (N.K.K.)

[3] Department of Physics, Yeungnam University, Gyeongsan 38541, Republic of Korea; amanohar.svu@gmail.com (A.M.)

[4] School of Chemical Engineering, Theranostic Macromolecules Research Center, Sungkyunkwan University, Suwon 16419, Republic of Korea; thambi@skku.edu (T.T.)

[5] Faculty of Applied Sciences, Ton Duc Thang University, Ho Chi Minh City, Vietnam. duonghaisang99@gmail.com (H.-S.D.)

* Correspondence: euesoon@kumoh.ac.kr (E.-S.J.); ehchoi@kw.ac.kr (E.H.K.).

† P.M., S.A., and A.M. contributed equally to this work.

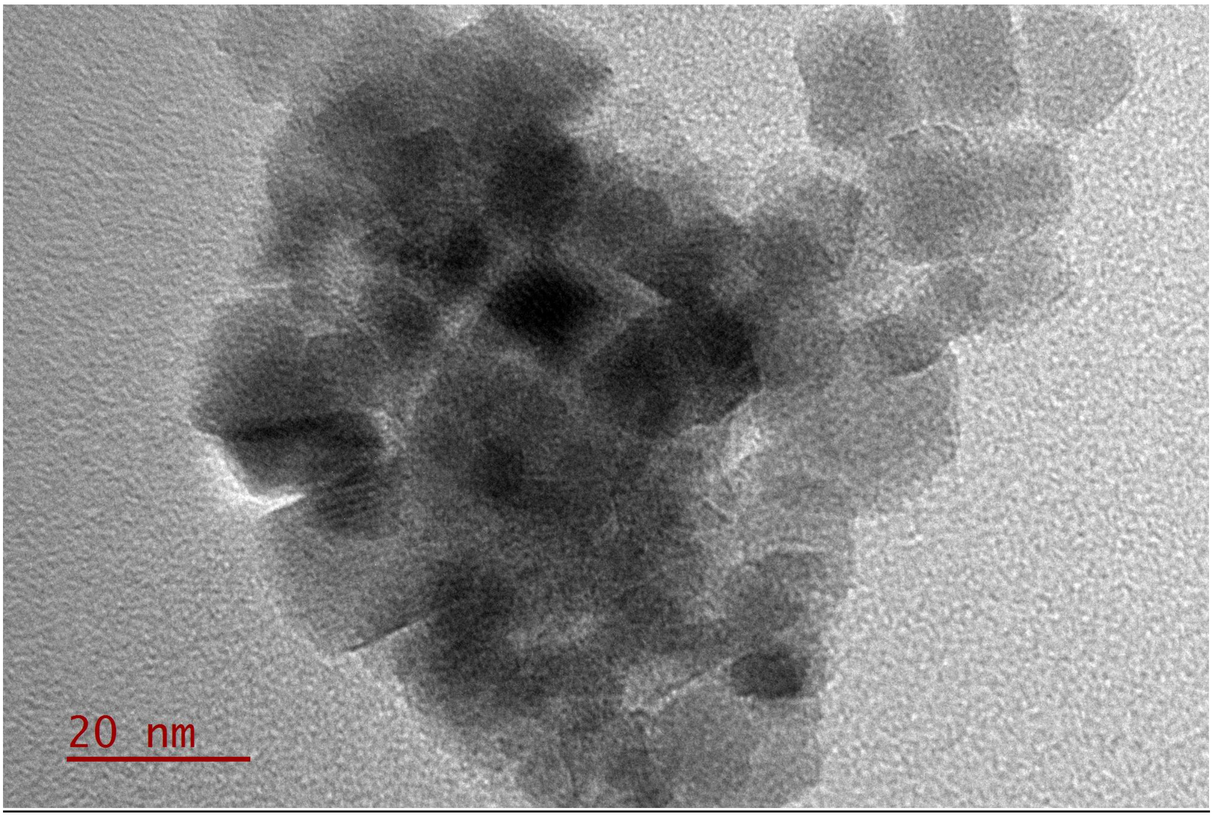

Figure S1. TEM image of Ca^{2+}-doped $MgFe_2O_4$ NPs.

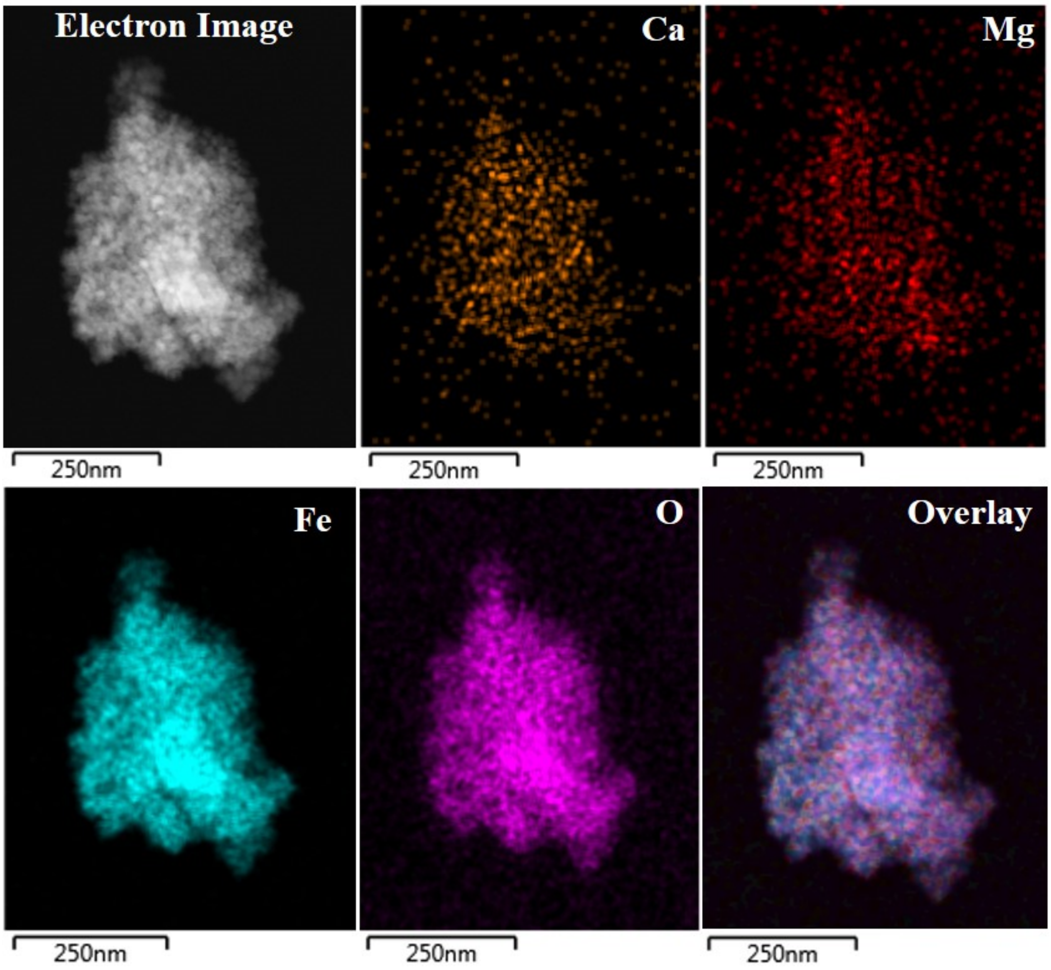

Figure S2. Cs-corrected FETEM with EDX mapping.

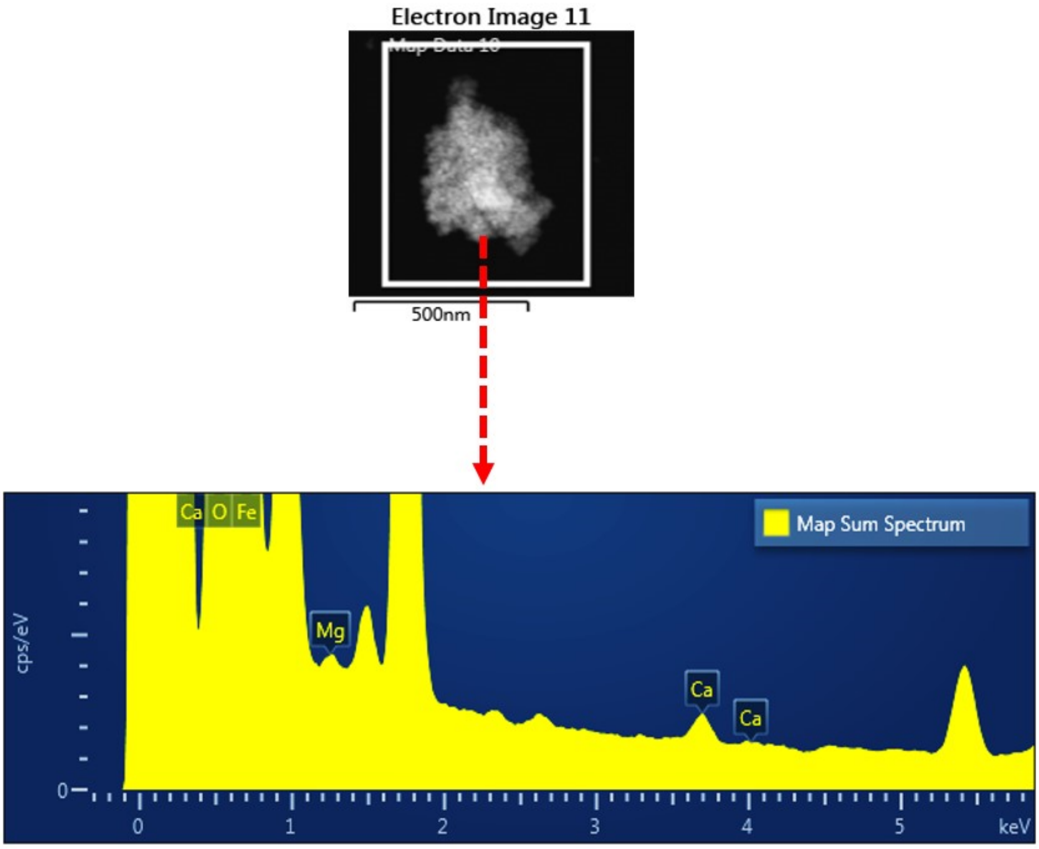

Figure S3. Cs-corrected FETEM with EDX spectroscopy.

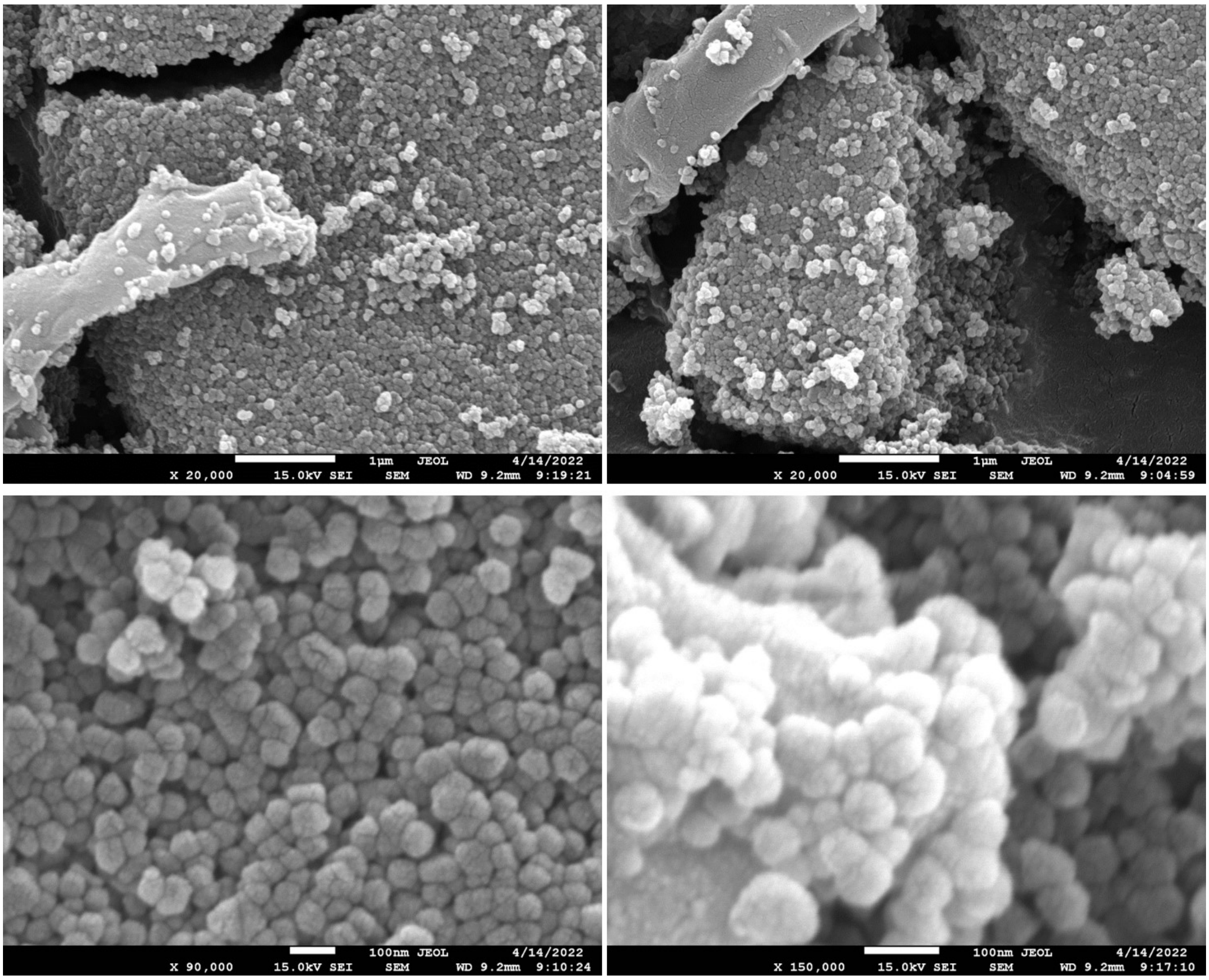

Figure S4. SEM images of Ca^{2+}-doped $MgFe_2O_4$ NPs.

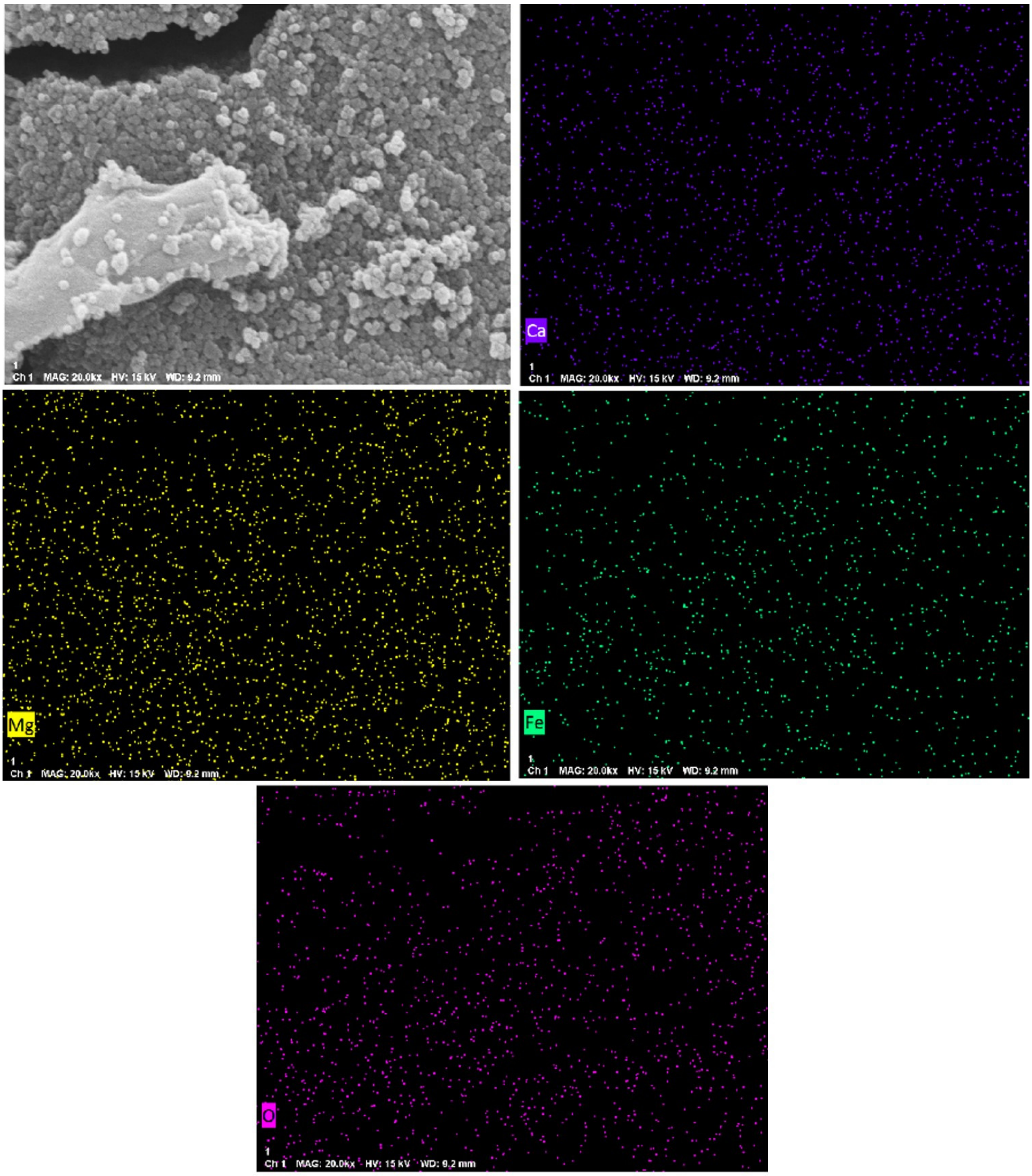

Figure S5. SEM with EDX mapping of Ca^{2+}-doped $MgFe_2O_4$ NPs.

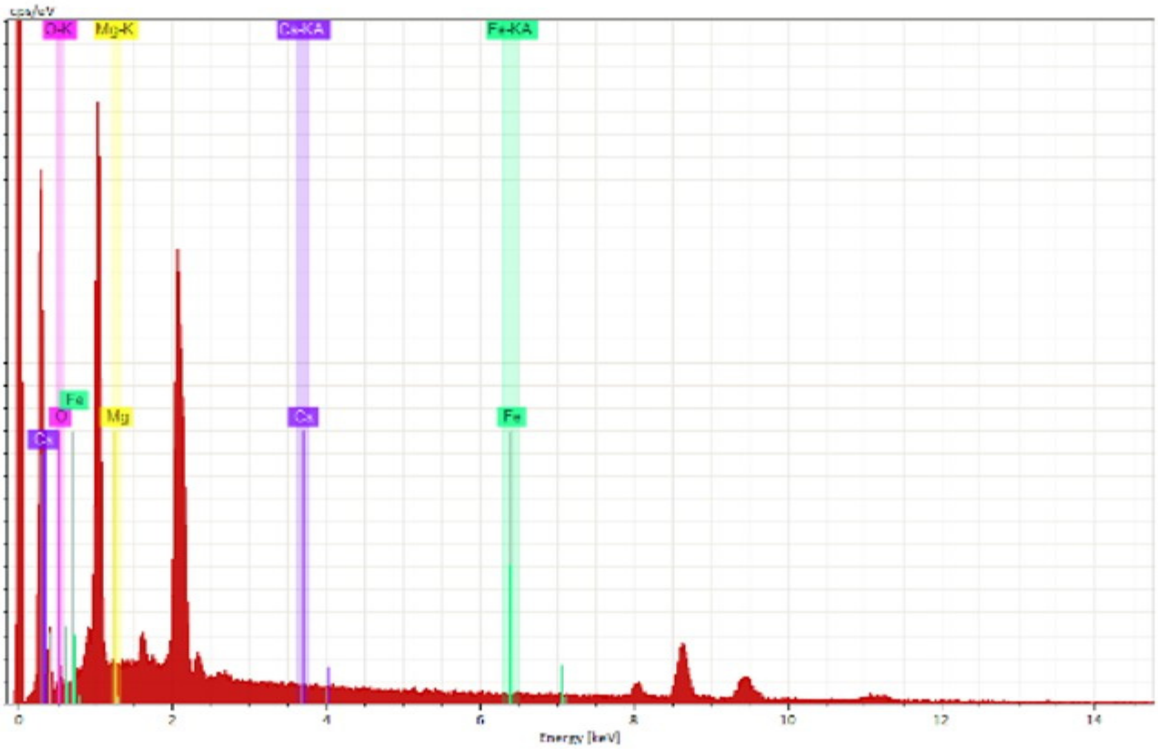

Figure S6. SEM-EDX spectra of Ca^{2+}-doped $MgFe_2O_4$ NPs.

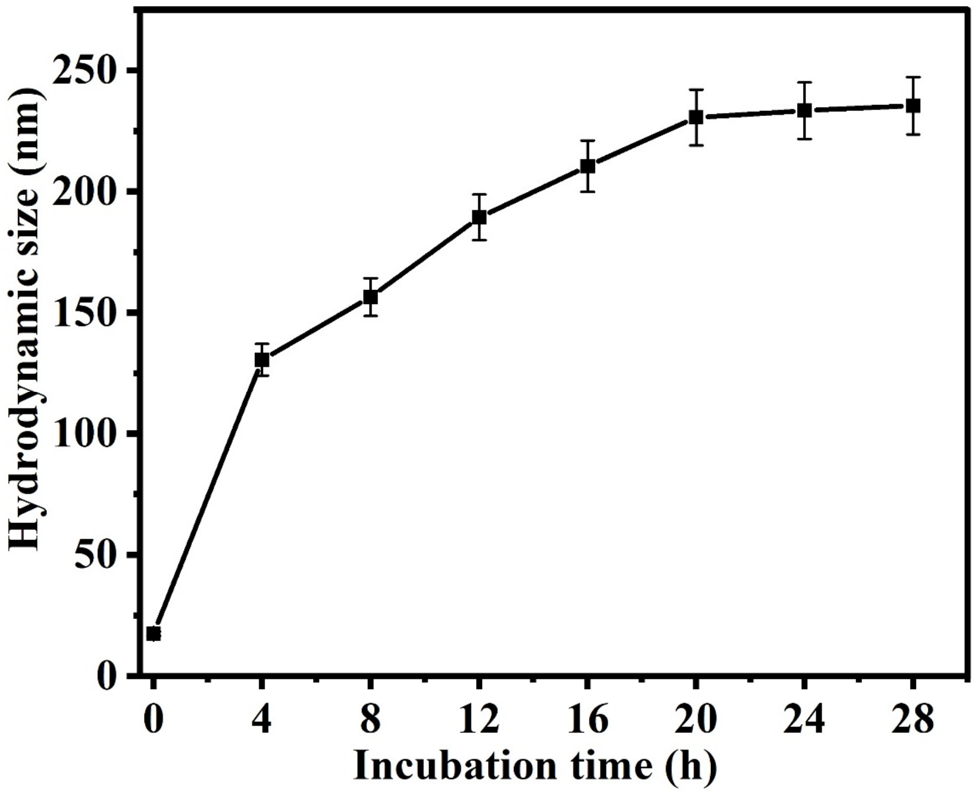

Figure S7. Hydrodynamic size of Ca^{2+}-doped $MgFe_2O_4$ NPs after incubation in RPMI medium supplemented with 10% FBS.

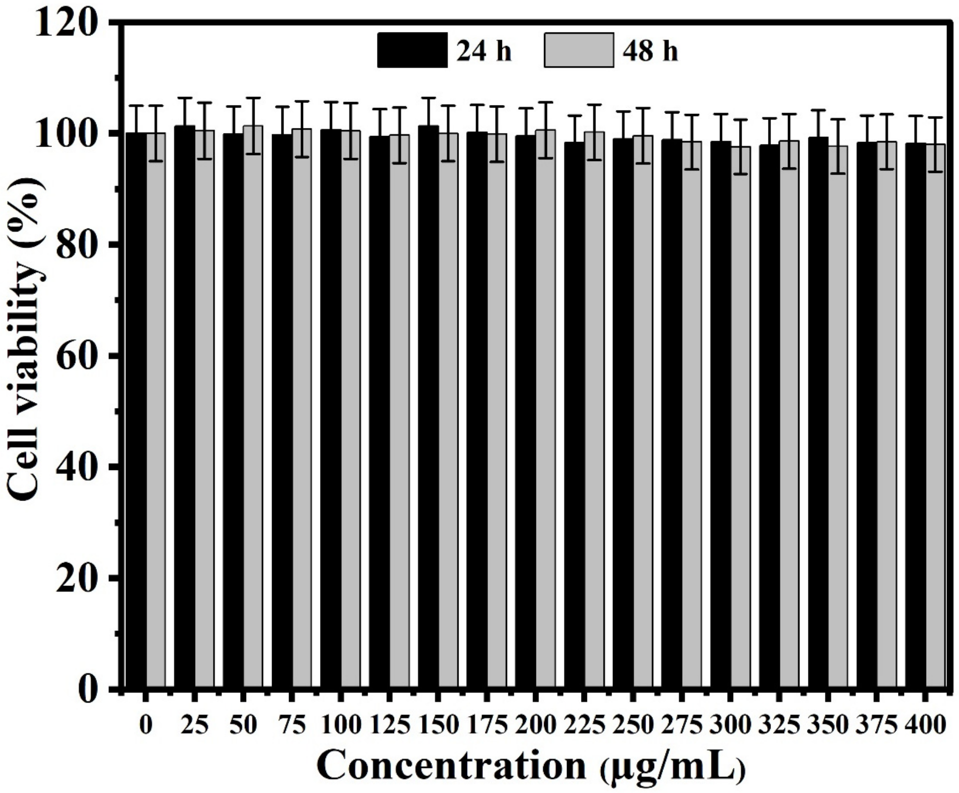

Figure S8. *In vitro* biocompatibility of Ca^{2+}-doped $MgFe_2O_4$ NPs at various concentrations on HDF cells after 24 h and 48 h.

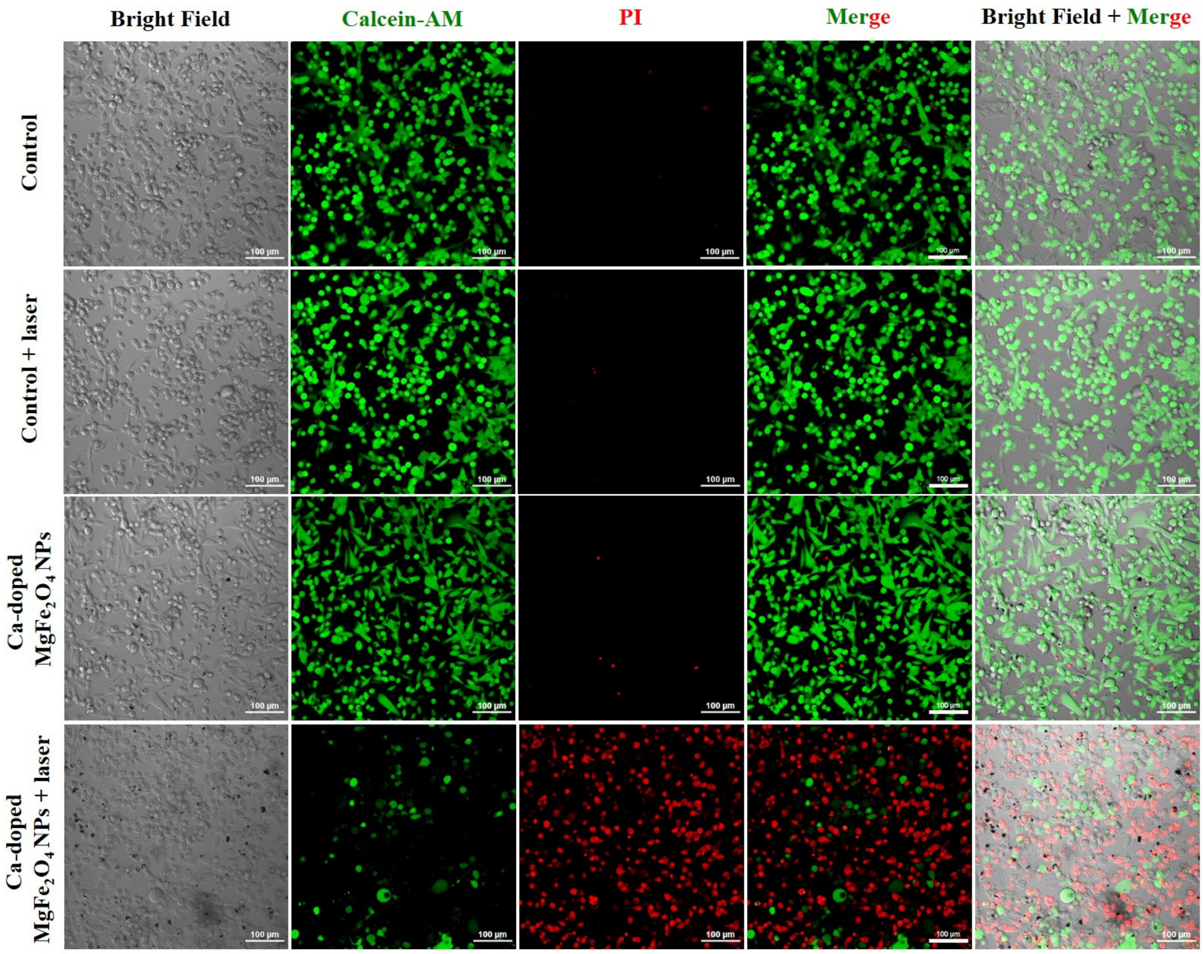

Figure S9. Confocal laser scanning microscope images of MDA-MB-231 cells treated with PBS only, PBS + laser irradiation, Ca^{2+}-doped $MgFe_2O_4$ NPs only, and Ca^{2+}-doped $MgFe_2O_4$ NPs + laser irradiation. Calcein-AM (green) and PI (red) were used to distinguish between live and dead cells (20 × magnification; scale bar: 100 μm).

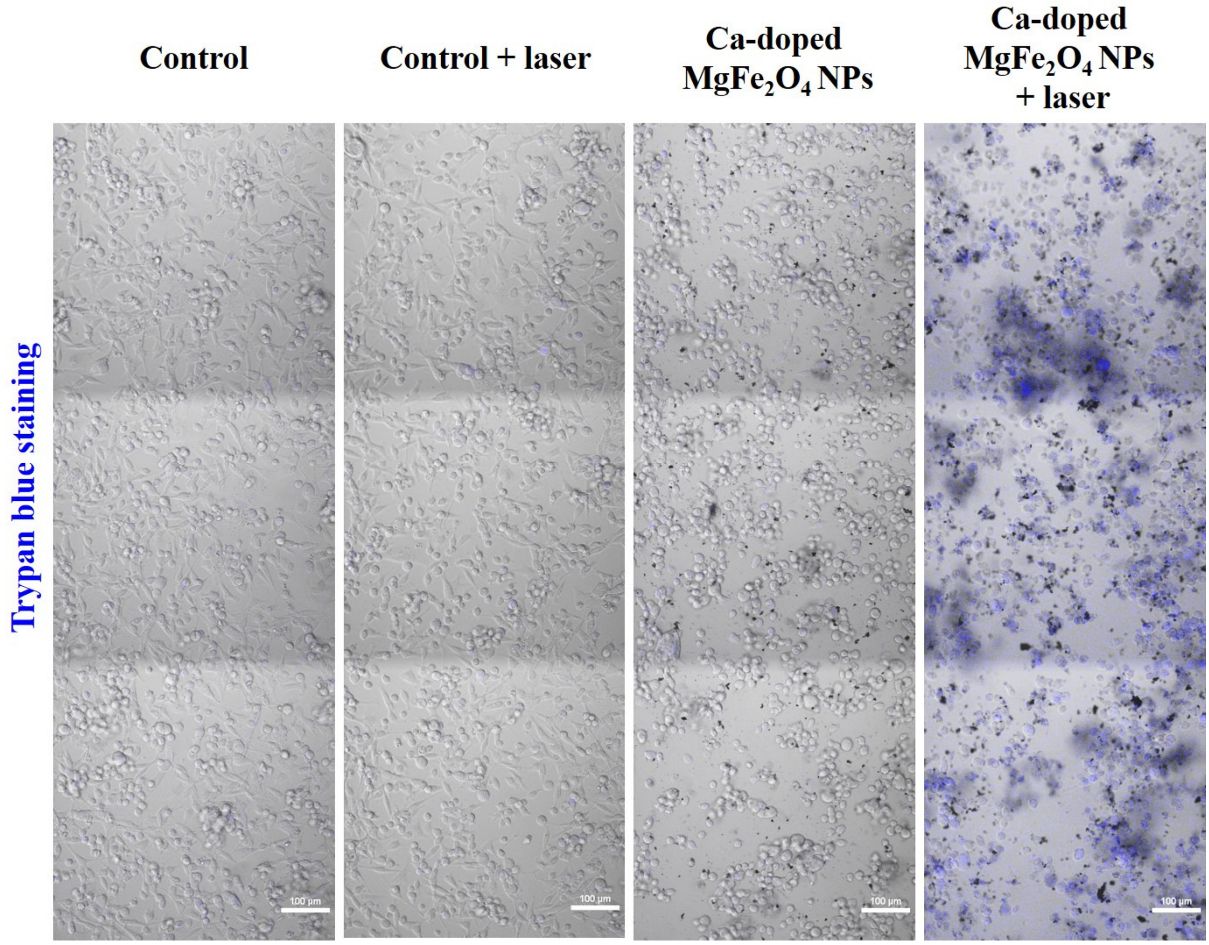

Figure S10. Optical images of trypan blue stained MDA-MB-231 cells treated with PBS only, PBS + laser irradiation, Ca^{2+}-doped $MgFe_2O_4$ NPs only, and Ca^{2+}-doped $MgFe_2O_4$ NPs + laser irradiation (20 × magnification; scale bar: 100 μm).

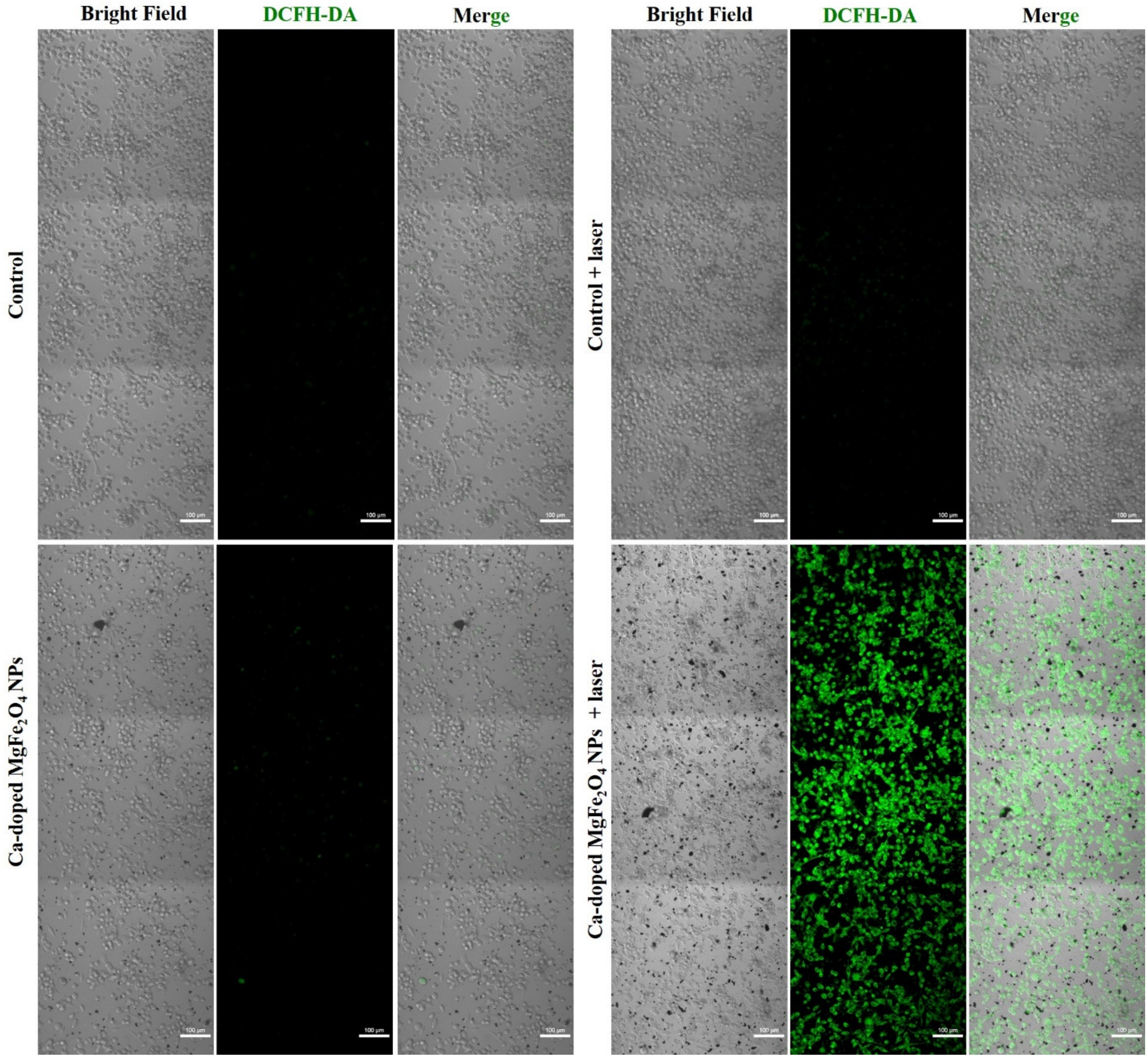

Figure S11. Confocal laser scanning microscopy images of DCFH-DA-stained MDA-MB-231 cells treated with PBS only, PBS + laser irradiation, Ca^{2+}-doped $MgFe_2O_4$ NPs only, and Ca^{2+}-doped $MgFe_2O_4$ NPs + laser irradiation, for determining the intracellular ROS level (20 × magnification; scale bar: 100 μm).

Table S1. Comparative study of the photothermal effect of Ca^{2+}-doped $MgFe_2O_4$ NPs and various ferrite NPs.

NPs	Cell line	*In vitro* biocompatibility test (%)	Laser	Temp (°C)	Photothermal conversion efficiency ((η)	*In vitro* PTT (%)	References
Ca^{2+}-doped $MgFe_2O_4$ NPs	MDA-MB-231 cells	94.5%	785 nm, 0.8 W/cm², 5 min	51.7 °C	30.12%	32.37%	
**Comparison of various ferrite NPs**							
$ZnFe_2O_4$ NPs	A549 cells	> 80%	808 nm, 2.0 W/cm², 10 min	69.0 °C	37.7%	26.9%	[1]
GO/$MnFe_2O_4$ NPs	HeLa cells	> 92%	808 nm, 0.5 W/cm², 10 min	43 °C	-	32.1%	[2]
Bismuth ferrite NPs (BFO NPs)	HepG2 cells	> 90%	808 nm, 1.0 W/cm², 10 min	52.4 °C	51.4%	32.3%	[3]
$MgFe_2O_4$ NPs	HePG2 cells	> 90%	808 nm, 1.0 W/cm², 4 min	45.7 °C	-	12.0%	[4]

References

1. Wang, K.; Yang, P.; Guo, R.; Yao, X.; Yang, W. Photothermal performance of MFe2O4 nanoparticles. *Chin. Chem. Lett.* **2019**, *30*, 2013-2016.
2. Yang, Y.; Shi, H.; Wang, Y.; Shi, B.; Guo, L.; Wu, D.; Yang, S.; Wu, H. Graphene oxide/manganese ferrite nanohybrids for magnetic resonance imaging, photothermal therapy and drug delivery. *J. Biomater. Appl.* **2016**, *30*, 810-822.
3. Yang, C.; Chen, Y.; Guo, W.; Gao, Y.; Song, C.; Zhang, Q.; Zheng, N.; Han, X.; Guo, C. Bismuth ferrite-based nanoplatform design: an ablation mechanism study of solid tumor and NIR-triggered photothermal/photodynamic combination cancer therapy. *Adv. Funct. Mater.* **2018**, *28*, 1706827.
4. Qiu, E.; Chen, X.; Yang, D.-P.; Regulacio, M.D.; Ramos, R.M.C.R.; Luo, Z.; Wu, Y.-L.; Lin, M.; Li, Z.; Loh, X.J. Fabricating Dual-Functional Plasmonic–Magnetic Au@ MgFe2O4 Nanohybrids for Photothermal Therapy and Magnetic Resonance Imaging. *ACS Omega* **2022**, *7*, 2031-2040.
